# IDSNN: Towards High-Performance and Low-Latency SNN Training via Initialization and Distillation

**DOI:** 10.3390/biomimetics8040375

**Published:** 2023-08-18

**Authors:** Xiongfei Fan, Hong Zhang, Yu Zhang

**Affiliations:** 1 State Key Laboratory of Industrial Control Technology, College of Control Science and Engineering, Zhejiang University, Hangzhou 310027, China; xffan@zju.edu.cn (X.F.); hongzhang99@zju.edu.cn (H.Z.); 2 Key Laboratory of Collaborative Sensing and Autonomous Unmanned Systems of Zhejiang Province, Hangzhou 310027, China

**Keywords:** spiking neural networks (SNNs), knowledge distillation, initialization, image classification

## Abstract

Spiking neural networks (SNNs) are widely recognized for their biomimetic and efficient computing features. They utilize spikes to encode and transmit information. Despite the many advantages of SNNs, they suffer from the problems of low accuracy and large inference latency, which are, respectively, caused by the direct training and conversion from artificial neural network (ANN) training methods. Aiming to address these limitations, we propose a novel training pipeline (called IDSNN) based on parameter initialization and knowledge distillation, using ANN as a parameter source and teacher. IDSNN maximizes the knowledge extracted from ANNs and achieves competitive top-1 accuracy for CIFAR10 (94.22%) and CIFAR100 (75.41%) with low latency. More importantly, it can achieve 14× faster convergence speed than directly training SNNs under limited training resources, which demonstrates its practical value in applications.

## 1. Introduction

Artificial neural networks (ANNs) have come a long way since their inception in the 1940s. From simple perceptrons to complex deep neural networks, ANNs have revolutionized the field of artificial intelligence. In recent years, with the help of deep learning algorithms, ANNs have achieved remarkable success in various applications, such as image and speech recognition, natural language processing, and game playing. However, ANNs rely heavily on computing resources, resulting in high energy consumption. Based on this, Spiking Neural Networks (SNNs) try to imitate biological mechanisms and offer energy-efficient advantages. By simulating the way neurons in the brain communicate through spikes or brief electrical pulses [1], SNNs can perform computations with minimal energy consumption [2,3]. This makes them well-suited for applications in low-power devices, such as IoT sensors or mobile devices, where energy efficiency is a critical factor. Furthermore, SNNs can also enable new computing paradigms, such as neuromorphic computing, which aim to mimic the brain’s ability to learn and adapt to new situations.

Limited by the non-differentiable nature of impulse signals, the backpropagation algorithm used in ANNs is not suitable for SNNs [4]. There are two training methods for SNNs: the conversion from ANN and the direct training based on surrogate gradients. The conversion method [5,6,7,8] transforms ANNs into SNNs by replacing the activation functions with spiking neurons and keeping the weights stable without extra training. However, this approach suffers from several drawbacks, including the high computational cost of training ANN, the inability to handle temporal information, and the high latency in inference. The direct training method, on the other hand, bypasses the conversion step and trains SNNs directly using surrogate gradients, which are simplified gradients that approximate the true gradients and allow for backpropagation-like training [9,10]. This approach can handle temporal information and overcome the problem of inference latency, making it more suitable for real-time applications. However, this method loses the performance gain of ANNs, and the resulting SNNs suffer from a significant decrease in accuracy. Given the huge potential of SNNs in the field of bionics, as well as their energy-saving advantages, it is crucial to investigate a training method that can conserve computational resources while maintaining excellent performance.

We recognize that the training methods for SNNs must adapt to the nature of spikes in order to fully realize their advantages. At the same time, ANNs already have mature training methods and excellent performance, with performance being positively correlated with model size. These achievements of large models are currently not available for SNNs. It is crucial to learn from these achievements to obtain SNNs, but we cannot rely solely on well-trained ANNs. In previous neural network training, the statistics-based Kaiming initialization has strong generalizability and is suitable for different models and datasets. However, its generalization comes at the cost of weaker specificity. Inspired by various distillation algorithms, we realized that the teacher model contains dataset-specific knowledge. When we use the teacher model’s parameters to initialize the SNN model, this knowledge-based (rather than statistics-based) initialization has particular advantages. In this paper, we show that SNNs can learn much from larger ANNs, through initialization and distillation to obtain excellent performance, and direct training—using a surrogate gradient ensures the ability to quickly infer and process temporal data. The main contributions of this work can be summarized as follows:

1. Based on the connection we established between ANNs and SNNs, we propose a knowledge-based method to enable initialization from larger to smaller models, or vice versa, which has good scalability.

2. Based on the initialization method we proposed between ANNs, SNNs, and knowledge distillation, we propose a new training method that fully utilizes the information from ANNs to directly train SNNs. Firstly, we trained a larger-scale ANN and initialized the target SNN through the parameter connection. Then, we used the ANN as a teacher to train the student SNN through the surrogate-gradient method.

3. We evaluated our method and achieved 75.41% and 94.22% top-1 accuracy on the CIFAR100 and CIFAR10 datasets, exceeding common direct training SNNs by 4.10% on CIFAR100 and 0.68% on CIFAR10. Moreover, our model made a greater contribution by significantly accelerating the convergence speed (**14×**) at the same time as achieving better accuracy.

## 2. Related Works

### 2.1. ANN-to-SNN Conversion

Considering that the training of ANNs is already very mature, converting well-trained ANNs to SNNs can reduce computation and memory usage. The conversion is based on the link between the transfer function of a spiking neuron and ReLU in ANNs. Ref. [11] analyzed the feasibility of conversion of ANNs to SNNs, providing a theoretical basis for the conversion. Moreover, weight normalization [12], soft-reset [13], and threshold balancing [7] were proposed to mitigate the errors caused by the conversion. However, the existing methods for converting ANNs with BN layers require large time-steps (100–1000) for inference, which results in significant latency. On the other hand, SNNs obtained in this way are only suitable for static data. The high latency and data constraints posed by these simple conversion methods hinder the realization of the biological advantages offered by SNNs. As a result, alternative approaches are needed to enable efficient training and inference of SNNs.

### 2.2. Directly Training SNNs

The main method to obtain deep SNNs directly is backpropagation with surrogate gradients. The reason why SNNs cannot be directly trained using the BP algorithm is because of the non-differentiable nature of spikes. Surrogate-gradient methods [9,10,14] use an approximate continuous function to replace the actual gradient of the spiking neurons. Due to the inaccuracy of activation functions, errors accumulate over time-steps and iterations, leading to poor accuracy of such methods. Additionally, several other approaches have been proposed to improve the accuracy of SNNs. For example, researchers have enhanced the ReLU activation function to evolve towards spiking neurons during the training process [15]. This allows the SNN to absorb some of the information that the ANN can learn. Another obvious way is to use knowledge distillation to introduce the output of an ANN as the supervisory information. LaSNN [16], for instance, used attention to bridge the information gap between ANNs and SNNs and proposed a three-stage layer-wise knowledge distillation training pipeline. The authors still used the conversion method to initialize the weights of the target SNN, resulting in the disadvantage of high latency. Furthermore, intermediate layer distillation has also been introduced to improve the accuracy of SNNs [17].

## 3. Materials and Methods

### 3.1. Spiking Neuron Model

The spiking neuron we used in this work is the Leaky Integrate-and-Fire (LIF) [18], which considers the current leakage of neurons more than IF does. In a multi-layer neural structure, the dynamic equations of a single LIF neuron are

(1) X(t)=W∗F(t−1)

(2) $$U\left(t\right)=\left\{\begin{array}{cc} U_{0}=X\left(t\right), & t=0\\ U\left(t-1\right)e^{-\frac{1}{\tau }}*\left(1-F\left(t-1\right)\right)+X\left(t\right), & t\neq 0 \end{array}\right.$$

(3) $$F\left(t\right)=F\left(U\left(t\right)\right)=\left\{\begin{array}{cc} 1, & U\left(t\right)>U_{th}\\ 0, & otherwise \end{array}\right.$$

where *t* denotes the timestamp, τ is the time coefficient, X(t) is the input current from the last neuron, *W* means the weight matrix between neurons, and F(t) is the output of the spiking neuron at timestamp *t*, which is 1 if activated and 0 otherwise. $U_{0}$ is the initial membrane potential at timestamp 0. U(t) denotes the hidden membrane potential, which continuously receives input current until the neuron is activated and reset to 0. $U_{th}$ is the activation threshold that determines whether or not the neuron is activated.

The step function in Equation (3) results in the non-differentiable characteristic. We used the surrogate gradient proposed in STBP [19]. We took a neighborhood of length 2∗lens on both sides of $U_{th}$, assuming that the membrane potential range where the spike fires was within this neighborhood, in order to calculate the surrogate gradient as follows:

(4) $$F^{\prime }\left(U\left(t\right)\right)=\left\{\begin{array}{cc} \frac{1}{2*lens}, & U_{th}+lens>U\left(t\right)>U_{th}-lens\\ 0, & otherwise \end{array}\right.$$

where lens represents half the length of the neighborhood mentioned above. Hence, both forward information transfer and back error propagation between spiking neurons can be carried out.

### 3.2. Connect ANNs and SNNs through Initialization

#### 3.2.1. The Relationship between Spiking ResNet and ResNet

Currently, research works  [14,20,21,22] typically utilize residual structures to obtain deep SNNs, just as in ANNs. By stacking residual blocks with different numbers, it is possible to achieve deep residual models of different sizes, such as ResNet18, ResNet34, ResNet50, ResNet101, etc. [23].

**Figure 1:**
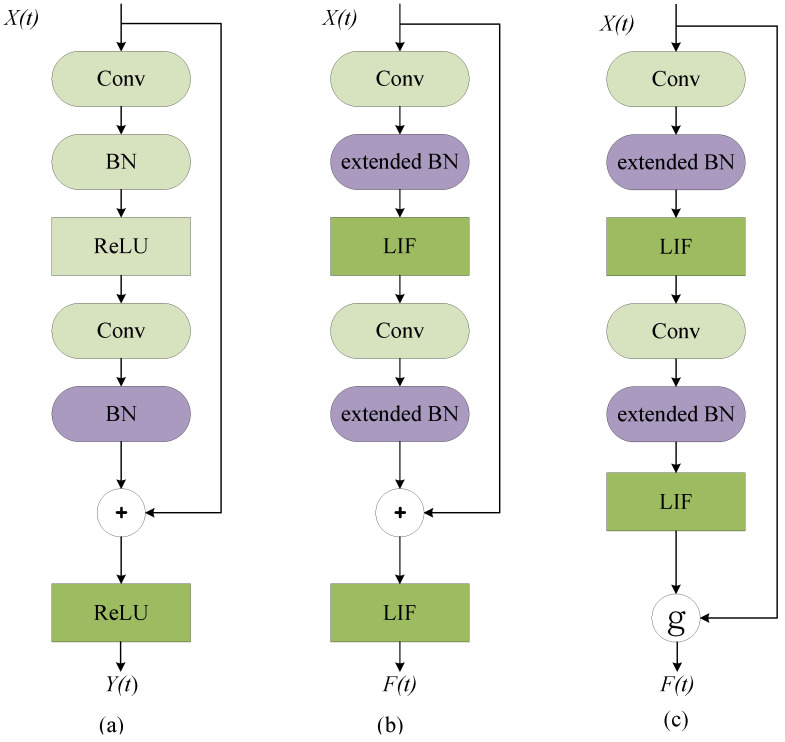
The structure of ResNet block and Spiking ResNet block. (**a**) Basic block in ANN ResNet. (**b**) Basic block in Spiking ResNet. (**c**) Basic block in SEW ResNet.

The residual block is the main component of ResNet. Figure 1a shows the basic block in ResNet. It consists of convolution layers, batch normalization layers, and activation functions. X(t) denotes the input from the last block at time-step t, and Y(t) denotes the output of this residual block. The basic block of Spiking ResNet used in [20,21,22] simply mimics the block in ANNs by replacing ReLU activation layers with spiking neurons (e.g., LIF), which is illustrated in Figure 1b. While Spiking ResNet suffers from the problem of vanishing or exploding gradient, [14] used Spike-Element-Wise block to mitigate this, as Figure 1c shows.

No matter the type of Spiking ResNet structure, we can find that it consists of convolution layers and BN layers, similar to the ResNet structure in ANNs. The only difference is that the activation function (such as ReLU) is replaced by spiking neurons (such as LIF).

Based on this observation, a natural idea is that we can use a pre-trained ANN model to obtain an SNN model, and the conversion method is one possible solution. The conversion method transfers the parameters of ANN to SNN and uses the link between the transfer function of a spiking neuron and ReLU to determine the activation of spiking neurons. As mentioned earlier, it suffers from high inference latency, which limits its practical value. We believe that the relationship between the parameters can be utilized, as they have highly similar convolution and BN layers. However, the non-parametric neuron activation cannot be determined using the ReLU activation function, as they represent two completely different encoding schemes. Based on this idea, we proposed an initialization-based method to exploit the parameter relationship between ANN and SNN.

#### 3.2.2. SNN Initialization through ANN

Although the parameterized layers are convolution and BN layers in both ANN and spiking residual blocks, the temporal and spatial properties of spiking neural networks require an additional time dimension in the input, resulting in structural differences between convolution and BN layers. Furthermore, taking inspiration from knowledge distillation, the benefits of initialization from a larger network outweigh those from a same-size network. Therefore, SNN initialization through ANN requires overcoming both model size and model structure issues in parameter initialization.

**Figure 2:**
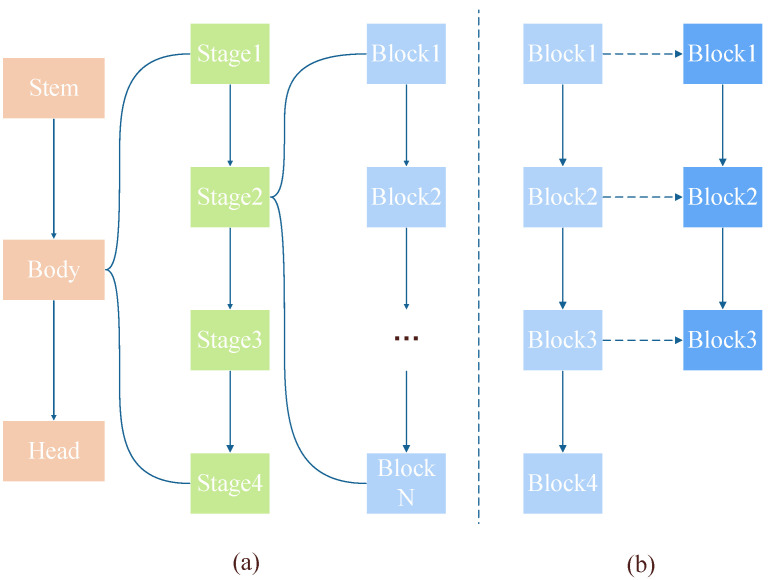
(**a**) The structure of ResNet. (**b**) The correspondence during initialization at block level.

We first focus on the initialization problem of model size (block level). In this work, we only discuss the ResNet model family. Figure 2a shows the structure of ResNet. The body of ResNet consists of four stages—every stage consists of different numbers of blocks—which create ResNet with different depths (e.g., ResNet18, ResNet34, etc.). We believe that every block has an impact on the blocks before and after it in the same stage, meaning that the order of blocks in the same stage contains spatial information about the model. As Figure 2b shows, when initializing parameters at the block level, we preserved this spatial relationship. As we used a larger ResNet to initialize the Spiking ResNet, the number of blocks in the ANN was greater than that in the SNN in the corresponding stage. Each block in the SNN could be initialized with the corresponding ANN parameters, maintaining the relative spatial relationship in the ANN.

**Figure 3:**
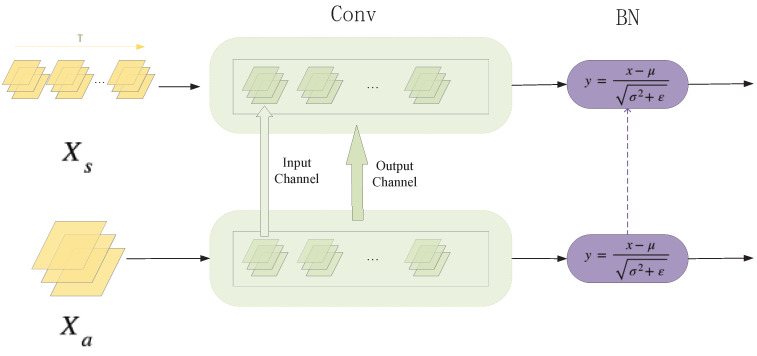
The initialization process in convolution and BN layers.

After completing initialization at the block level, we turned our attention to the issue of model structure (convolution and BN layers in the block). As Figure 3 shows (for the sake of convenience, the batch dimension is omitted), set $X_{s}\in R^{B\times T\times C\times H\times W}$ denotes the input of the spiking residual block, while $X_{a}\in R^{B\times C\times H\times W}$ denotes the input of the residual block. The *T* dimension of $X_{s}$ is a repetition of the input sample to simulate the time-step of the spiking neural networks. When using a residual block of the larger source ANN to initialize that of the target SNN, we met differences in the channel dimension of convolution layers. To be specific, a convolution layer contained many kernels (called output channels), and one kernel contained many channels to process input data (called input channels). Firstly, in the output channel dimension, we performed loop interpolation based on the convolution kernel to ensure that each SNN convolution kernel had a corresponding source of ANN convolution kernel parameters. Then, in the input channel dimension, we performed the same loop interpolation, but based on the channel of kernels to complete the initialization between convolution kernels. At this point, we had completed the initialization of the convolution layers in a residual block.

(5) $$y=\frac{x-\mu }{\sqrt{\sigma^{2}+\epsilon }}$$

As shown in Equation (5), batch normalization computes an output *Y*, which normalizes input *X* using per-channel statistics μ, $\sigma^{2}\in R^{C}$. Here, *x* is a feature with a shape of (T,C,H,W). Generally speaking, the normalization operation of the batch normalization layer is followed by a channel-wise affine transform. It has two parameters: weight and bias. Therefore, there are four parameters in the batch normalization layer: mean, variance, weight, and bias. They are all one-dimensional vectors, also with a size of *C* extended in time dimension. These four one-dimensional parameters can be converted from the ANN model to the SNN model directly.

In this section, we have discussed how to address the initialization problem for model size and different model structures. We are now able to use a larger ANN to initialize the target SNN for training preparation.

### 3.3. IDSNN

We have initialized the SNN with the parameters of an ANN, which allows it to absorb some knowledge from the ANN before training. In this section, we discuss how to fully leverage ANN through knowledge distillation during the training process, as well as the complete training pipeline.

#### 3.3.1. ANN-to-SNN Distillation

**Figure 4:**
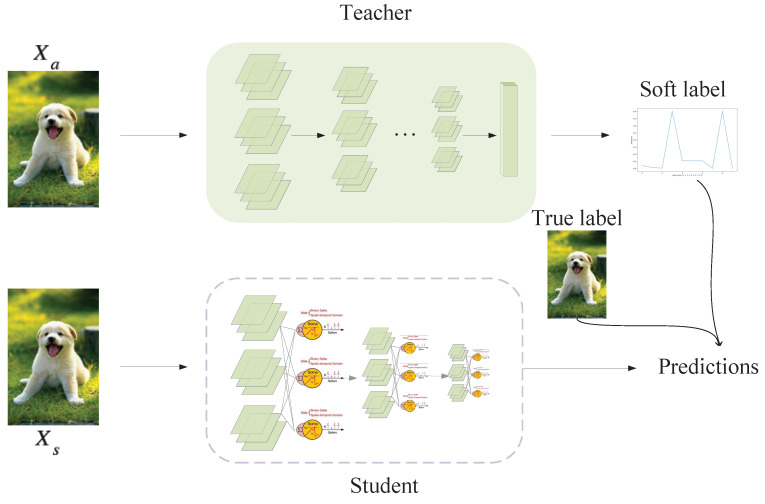
Schematic illustration of distillation training.

Just as in ANN, there has been some exploration of knowledge distillation in SNNs, such as distilling spikes from a large SNN to a small SNN  [24] and distilling feature-based knowledge from a well-trained ANN to an SNN  [17]. However, the approaches mentioned above either selected an inappropriate teacher model or complicated the distillation process without bringing much improvement in performance. As proven in ANN, the simplest knowledge distillation [25] method may be the most effective. Furthermore, because spikes and analog formats are two different information encoding methods, even if the ANN teacher and SNN student have similar network structures, there are differences in how the intermediate layers encode data. Based on these two considerations, we abandoned the intermediate-layer complex distillation and proposed a concise and efficient distillation method suitable for SNNs, as Figure 4 shows. While training, the parameters of the teacher model were fixed, and we fed the same sample into the teacher and student models. The proposed method used response-based distillation to guide the student SNN training using the output of the softmax function in the teacher ANN. The soft label contained more knowledge because it included the probability distribution information of the sample on all class categories relative to the true label, as shown in Equation (6):

(6) $$q_{i}=\frac{e^{Z_{i}/T}}{\sum_{j}e^{Z_{j}/T}}$$

where $Z_{i/j}$ denotes the probability that a sample belongs to a certain class and *T* determines the significance level of the probability distribution, which is related to the difficulty level of learning knowledge conveyed through distillation. Combining soft labels with richer information and absolutely correct true label, we adopted the following loss function:

(7) $$L_{kd}=KL\left(softmax\left(Q_{s}/T\right),softmax(Q_{t}/T)\right)$$

(8) $$L_{total}=CrossEntropy\left(Q_{s},y_{true}\right)+\alpha *T^{2}*L_{kd}$$

where $y_{true}$ denotes the true labels, and $Q_{s}$ and $Q_{t}$ are the outputs of student SNN and teacher ANN. The total loss is obtained by summing two-part loss and using α to indicate the importance of the two learning targets.

#### 3.3.2. IDSNN Training Process

The entire IDSNN training process is divided into three steps, as illustrated in Algorithm 1. Firstly, it trains a larger teacher ANN using a conventional ResNet. Here, we used ResNet34 as the ANN teacher.

Secondly, it uses the parameters of the trained ANN to initialize a smaller-sized student SNN, according to the method described in the previous section. This step mainly focuses on the parameters of the convolution layer and the BN layer, while keeping the parameters of the fully connected layer randomly initialized. The firing of the spiking neurons is determined by the next step of training. We used Spiking ResNet18 as the target SNN, without any trick in structure design, to verify the effectiveness of our training method.

After finishing the training of the teacher model and initialization, all the preparation work for training has been completed. We used the method of surrogate gradient and backpropagation algorithm to train target SNN with teacher ANN distillation. The response-based distillation scheme was adopted for transferring the knowledge of the teacher ANN to the student SNN. The distillation strategy and loss function design are described in the above subsections.
**Algorithm 1** Training pipeline**Require:** Pre-trained ANN $M_{t}$, target SNN $M_{s}$, input samples *X*, true label $y_{true}$**Ensure:** $M_{t}$ and $M_{s}$ are both ResNet-based  # initialization  # forward propagation  **for** t=1 to *T* **do**     X(t)←PoissonEncoder(X(t))     **for** l=1 to L−1 **do**       **if** t==1 **then**          $F_{l-1}^{t}=X\left(t\right)$       **end if**       # receive input from the previous layer and accumulate membrane potential       $U_{l}^{t}=U_{l}^{t-1}*e^{-\frac{1}{\tau }}*\left(1-F_{l}^{t-1}\right)+F_{l-1}^{t}$       $F\left(t\right)=F\left(U_{l}^{t},U_{th}\right)$     **end for**  **end for**  # calculate total loss $L_{total}$$\;\;L_{total}=CrossEntropy\left(Q_{s},y_{true}\right)+\alpha *T^{2}*L_{kd}$  # Backward Propagation  **for** t=T to 1 **do**     **for** l=L−1 to 1 **do**       $\frac{dL_{total}}{dU_{l}^{t}}=\frac{dL_{total}}{dF_{l}^{t}}\frac{dF_{l}^{t}}{dU_{l}^{t}}=\frac{dL_{total}}{dF_{l}^{t}}\times \frac{1}{2U_{th}}$     **end for**  **end for**

## 4. Results and Discussion

In this section, we evaluate the proposed IDSNN method on two benchmark datasets, CIFAR10 and CIFAR100. To further show the advantage of convergence speed, we tested it with various optimization strategies. Moreover, we conducted ablation experiments to validate the effectiveness of the initialization module and distillation module.

### 4.1. Experiments Setting

For both teacher ANN and target SNN training, we set the batch size as 64 and employed an SGD optimizer. The training epoch was set as 200, the learning rate was 0.05, the momentum was 0.9, and the weight decay was 0.0001. The teacher ANN was trained from scratch. In target SNN training, the time-step was set to four for fair comparison and six for better performance. All performances were evaluated on four NVIDIA GeForce RTX 2080ti GPUs.

### 4.2. Performance Comparison with Other Methods

In all experiments, we trained a ResNet34 as the teacher to initialize and knowledge distill. We validated the performance of IDSNN on the CIFAR10 and CIFAR100 datasets and compared with conversion-based methods, direct training methods, and knowledge distillation-based methods. Results are shown in Table 1 and Table 2. On the CIFAR10 dataset, the ResNet18 structure trained with our proposed method achieved a test accuracy of 94.03% using a time-step of four with the ResNet34 teacher model. IDSNN outperformed all other methods in terms of accuracy using the same or smaller time-step (T=4). Compared to three of the latest and best-performing distillation-related works from 2023, under the condition of using time-step of four (i.e., less than the three other works), our method achieved 0.62%, 2.81%, and 8.6% higher accuracy than KDSNN [17], LaSNN [16], and [26], respectively. In particular, when using ANNs that had the same accuracy (95.10%) as teachers, the accuracy of IDSNN was 0.62 higher than KDSNN. This indicates that our method is able to extract more useful knowledge from ANNs compared to others. When the time-step was increased to six, this further improved the accuracy to 94.22%.

On CIFAR100, the superiority of IDSNN was further demonstrated because, in general, a larger dataset requires higher classification capabilities from the model. It could achieve an accuracy of 75.24 using a time-step of four and 75.41 using a time-step of six. Compared with the KD training method [26], IDSNN also achieved better performance (75.24 vs. 74.42) using a shorter time-step (four vs. five). Compared to direct SNN training method Dspike [27] and Real Spike [29], IDSNN surpassed them by 1.89 and 10.37, respectively, in terms of accuracy when the time-step was four. This is because our initialization and distillation strategies largely compensated for the error accumulation caused by the surrogate gradient, thus achieving better accuracy. As for the methods (SNN Calibration [11], COS [30], Parameter Calibration [28]) based on conversion, they need an extremely large time-step to achieve good performance. Our proposed method has a crushing advantage both in time latency and absolute accuracy. When using an ANN that achieved an accuracy of 81.51%, Parameter Calibration [28] could only achieve an accuracy of 71.86%. This means that methods based solely on conversion cannot fully obtain information from ANNs within a short time delay. In comparison, IDSNN requires lower demands on ANNs and can enable SNNs to achieve better performance.

**Table 2:** Accuracy comparison with other SNN training methods on CIFAR100.

Model	Training Method	ANN	SNN	ANN Acc (%)	SNN Acc (%)	Time-Step
[31]	KD training	–	VGG16	–	74.42	5
Dspike [27]	SNN training	–	ResNet18	–	73.35	4
Real Spike [29]	SNN training	–	ResNet20	–	64.87	4
SNN Calibration [11]	Conversion	–	ResNet20	–	72.33	16
COS [30]	Conversion	–	ResNet20	–	70.29	32
Parameter Calibration [28]	Conversion	–	ResNet20	81.51	71.86	8
**IDSNN**	Hybrid training	ResNet34	ResNet18	77.26	75.24 75.41	4 6

### 4.3. Ablation Experiments

To verify the effects of the initialization module and distillation module, we conducted an ablation experiment on both the CIFAR10 and CIFAR100 datasets. As shown in Table 3, when not using any of our methods, the SNN of the ResNet18 structure only achieved 93.54% on CIFAR10 and 71.34% on CIFAR100. When using ResNet34 to initialize Spiking ResNet18 based on the relationship between the structures of the two, the performance was improved to 93.64% and 74.47%. When applying knowledge distillation to the baseline, the performance was 93.98% and 73.71%. Both initialization and knowledge distillation have a significant impact on improving accuracy, especially on the CIFAR100 dataset. This further confirms that the current direct training methods for SNNs have significant limitations on larger-scale datasets, and utilizing information from ANNs can be helpful. Through combining initialization and Knowledge distillation, IDSNN achieved the best performance, with 94.22% on CIFAR10 and 75.41% on CIFAR100. Using both modules simultaneously is more effective than using either module alone, which indicates that the information obtained from ANNs by the two modules does not overlap. On CIFAR10, the performance improvement (0.68) brought by two modules even exceeded the sum (0.10 + 0.44 = 0.54) of the improvements achieved by each module used separately.

### 4.4. Convergence Speed Experiments

In the previous experimental section, we changed the learning rate every 60 epochs in order to ensure that the model was sufficiently trained. The previous text explains the superior performance of IDSNN under this experimental condition. However, a larger learning rate interval means a greater training cost. An excellent model should not only ensure outstanding absolute performance but also be able to use limited training resources and achieve good performance within fewer training epochs.

**Figure 5:**
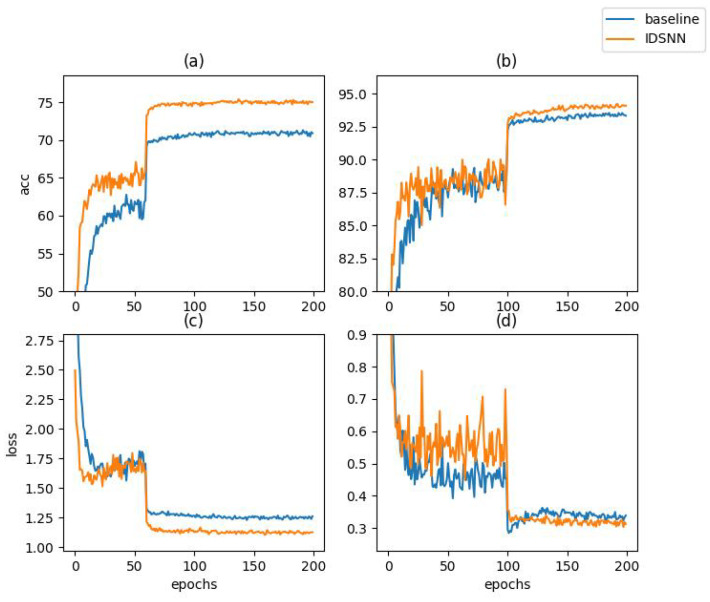
The comparison of loss and accuracy between IDSNN and baseline. (**a**) The accuracy on CIFAR100. (**b**) The accuracy on CIFAR10. (**c**) The loss on CIFAR100. (**d**) The loss on CIFAR10.

We changed the learning rate decay interval to 20 and 10 and compared the training performance of the baseline and IDSNN, as shown in Table 4. When the learning rate interval was 60, our proposed method had higher accuracy and faster convergence speed than the baseline, achieving 75.41% in epoch 138, while the baseline achieved 71.31% in epoch 192. When the learning rate interval decreased to 20, we reached the best accuracy 74.45% (only decreased by 0.96%) at epoch 54, while the baseline only reached 68.96 (decreased by 2.35%) at epoch 74. In more extreme cases, the learning rate interval was set to 10. Our proposed method (71.37%) only took 14 epochs to perform better than the baseline (71.31%) under sufficient training (192 epochs). This means that we could achieve the same or even higher accuracy at nearly 14 times the convergence speed. We chose the classical distillation and also conducted convergence speed experiments. Undoubtedly, distillation algorithms have some advantage over direct training algorithms in absolute accuracy. However, we noticed that, after changing the learning rate interval to 20 and 10, the accuracy dropped by 3.36% and 7.69%, respectively, which is even greater than the drops in the baseline (2.35% and 7.36%). Conversely, for IDSNN, the drops were 0.96% and 2.39%, and IDSNN required fewer iterations to reach the same accuracy level. The reason is that distillation algorithms provide more precise gradient updates during each backpropagation, which increases the accuracy upper bound and approximates a linear expansion of the convergence process. When the learning rate interval is reduced due to limited training resources, the insufficient convergence under the previous learning rate stage affects the next stage’s training, leading to a severe drop in accuracy. In comparison, IDSNN simultaneously raises the lower and upper bounds of the accuracy, shifting the entire training process towards higher accuracy. IDSNN can achieve the same or even better results with fewer iterations, and the smaller accuracy loss demonstrates stronger training robustness. Figure 5 shows the advantages of IDSNN in terms of convergence speed and accuracy.

## 5. Conclusions and Future Work

In this paper, we established a connection between ResNet and Spiking ResNet at the parameter initialization level and proposed a novel training pipeline based on knowledge distillation (IDSNN). We took full advantage of the knowledge of larger teacher ANNs to train SNNs with initialization and distillation. Experiments on CIFAR100 and CIFAR10 showed that we combined the advantages of direct training methods and conversion methods to achieve competitive performance (94.22% and 75.41%, surpassing most of the latest methods) at small time-steps (i.e., four, six). Both modules of our proposed method had a significant effect on improving the effectiveness. Furthermore, convergence speed experiments demonstrated its ability to converge quickly (14× faster) under limited training resources.

The focus of this work is the efficient training of SNN universal modules, which can better achieve downstream computer vision tasks by overlaying various classification heads. The biomimetic and energy-saving features of SNNs make this work more suitable for visual perception tasks on mobile robots. The limitation of this work lies in its applicability to only CNN networks, while the transformer structure is gaining prominence in the field of computer vision. For future work, we will explore training methods suitable for transformers and integrate training methods for both structures into a unified training framework.

## Figures and Tables

**Table 1:** Accuracy comparison with other SNN training methods on CIFAR10.

Model	Training Method	ANN	SNN	ANN Acc (%)	SNN Acc (%)	Time-Step
KDSNN [17]	KD training	Pyramidnet18	ResNet18	95.10	93.41	4
LaSNN [16]	KD training	–	VGG16	–	91.22	100
[26]	KD training	–	6Conv+2FC	–	85.43	8
Dspike [27]	SNN training	–	ResNet18	–	93.66	4
STBP-tdBN [22]	SNN training	–	ResNet19	–	92.92	4
QCFS [5]	Conversion	–	ResNet18	96.04	90.43	4
Parameter Calibration [28]	Conversion	–	ResNet20	96.72	92.98	8
**IDSNN**	Hybrid training	ResNet34	ResNet18	95.10	94.03 94.22	4 6

**Table 3:** Ablation study.

Model	Initialization	KD	ANN Teacher	Acc. on CIFAR10 (%)	Acc. on CIFAR100 (%)
ResNet18	✗	✗	–	93.54	71.31
	✓	✗	ResNet34	93.64 (↑0.10)	74.47 (↑3.16)
	✗	✓	ResNet34	93.98 (↑0.44)	73.71 (↑2.40)
	✓	✓	ResNet34	94.22 (↑0.68)	75.41 (↑4.10)

**Table 4:** Convergence experiments on CIFAR100.

		LR Strategy	[60, 120, 160]	[20, 40, 60]	[10, 20, 30]
	Acc (%)				
Model					
baseline			71.31(e192)	68.96(e74)	63.95(e85)
K-L divergence distillation			73.71(e195)	70.35(e49)	66.02(e37)
**IDSNN**			75.41(e138)	74.45(e54)	73.02(e57) 71.37(e14)

## Data Availability

The software and data presented in this study are openly available at https://github.com/fei015/IDSNN (accessed on 15 July 2023).

## Abbreviations

The following abbreviations are used in this manuscript:

ANNs	Artificial neural networks
SNNs	Spiking neural networks
BN	Batch normalization
LIF	Leaky Integrate-and-Fire
STBP	Spatio-temporal back propagation
SGD	Stochastic gradient descent
